# Influence of gender identity on the adoption of religious-spiritual, preventive and emotion-focused coping strategies during the COVID-19 pandemic in Pakistan

**DOI:** 10.1080/07853890.2023.2291464

**Published:** 2023-12-17

**Authors:** Saeed Ahmad, Sara Rizvi Jafree

**Affiliations:** a Department of Sociology and Anthropology, Utah State University, Logan, UT, USA; b Department of Sociology, Forman Christian College University (FCCU), Lahore, Pakistan

**Keywords:** Religious-religious coping, gender identity, pandemic, COVID-19

## Abstract

**Background:**

Academic research has highlighted the gendered impacts and amplifications of gender disparities of COVID-19. Traditionally, Pakistan is a patriarchal society, where it is a parenthood norm to socialize specific gender social roles.

**Objectives:**

The current research asserts that these normative gender roles may influence individuals throughout their life course, even during the COVID-19 pandemic. Therefore, the present study explored the influence of gender identity in adopting different coping strategies such as religious-spiritual, preventive, emotion-focused and non-constructive coping against the COVID-19 pandemic.

**Methods:**

Due to the lockdown in various areas of Pakistan, data were collected through an online questionnaire using Qualtrics. In a cross-sectional study, 955 respondents completed responses. Factors analysis and reliability analysis were run to ensure the scales’ reliability, validity and robustness for different coping strategies. Multivariate linear regression analysis was used to find model fitness.

**Conclusions:**

For theoretical explanation, the current study used *social role theory* that argues that each gender benefits differently from distinct coping behaviours. The findings highlighted that women were more likely to adopt most coping strategies, with the most significant difference in religious-spiritual coping and preventative coping strategies even in the presence of control variables such as level of education, household monthly income, family structure, marital status and family size. There was no gender difference in adopting non-constructive strategies. The empirical evidence suggested that females might be at an increased risk of stress due to the burden of unbalanced household-based social norms and care responsibilities. The current research also expanded the base of coping to religious-spiritual coping, emotion-focused coping and non-constructive coping.

## Key messages

- In Pakistan, higher adoption of coping strategies among women compared to men (such as religious-spiritual coping, preventative coping, emotion-focused coping, etc.) has raised serious concerns related to increased risk of stress and emotional fatigue among females in the country due to their coping behaviours.
- More research is needed to develop and support women’s active and productive role within the household to promote public health and positive coping strategies for families.

## Introduction

The COVID-19 pandemic had posed some serious psychological issues (such as stress, mental well-being, etc.) as a global public health problem [1]. These psychological issues also might have been affected by occurrence and spreading of illness, mortality and many policy-based responses (such as lockdown, social isolation, quarantine, etc.) to the COVID-19 pandemic. This situation resulted in the adoption of different coping strategies such as preventive coping, emotion-focusing coping, religious-spiritual coping, etc. among the public. Literature before the COVID-19 pandemic showed higher gender differences in the adoption of coping strategies [2,3]. These research patterns existed during COVID-19 pandemic as well. Some scholars argue that women have been impacted emotionally more than men [4,5]. It may be because the psychological impact of trauma which is more severe for women than for men [6].

In addition, for women specifically, there have been more significant burdens imposed by the pandemic on the home front, ranging from working from home remotely, supervising children’s home-based learning, and adding domestic duties for household management and care work [7]. It is also true that during a pandemic or sickness of family members, the burden of care, prevention, hygiene and recovery falls on the shoulders of women [8]. Recent literature also suggests that women are facing increased domestic and intimate partner violence since the COVID-19 pandemic, subsequent lockdowns [9] and the amplification of gender inequalities in the labour market [10].

Though the COVID-19 pandemic has changed the lives of everyone, for the reasons outlined above, the impact on and response of women may be different from that of men [11–13]. When women face health crises and social challenges, they adopt diverse coping strategies to retain balance in their lives and continue their roles as care providers [14]. This dominant care may compel women to adopt coping strategies to overcome stress and anxiety [15]. Literature has commented on different aspects of coping with gender differences, including religious-spiritual coping, preventive coping, emotion-focused coping and non-constructive coping [16–19].

As we go through the pandemic, it is essential to investigate the coping strategies adopted by women and compare the differences between the two genders, as these findings can serve as guidelines in the future. Academic literature has suggested that adopting a gender lens to study the effects of the COVID-19 pandemic is essential, such as the actions put into place at the global, national and community levels [20]. In addition, it is crucial to explore gender differences in specific health behaviour because gendered health behaviour variations may contribute to greater severity and fatality during the COVID-19 pandemic. The World Health Organization [21] has also recommended that countries incorporate a focus on gender into their COVID-19 responses to ensure that public health policies and measures to curb the epidemic take account of gender and how it interacts with other areas of inequality. Scholars agree that more research is needed to understand the gender variations in the adoption of coping strategies during the coronavirus pandemic [17,20,22]. In lieu of this, this study aims to explore gender-based differences in the adoption of different coping strategies, including (i) religious-spiritual coping, (ii) preventive coping, (iii) emotion-focused coping and (iv) non-constructive coping during the COVID-19 pandemic.

## Literature review

### Pakistani background

Pakistan is a traditionally patriarchal society, where it is a parenthood norm to socialize specific social roles for the children based on genders. So, the current research asserts that these normative gender roles may influence individuals throughout their life course. Therefore, even during the COVID-19 pandemic, life chances and choices will be affected by gender-based socialization.

**Figure 1.**
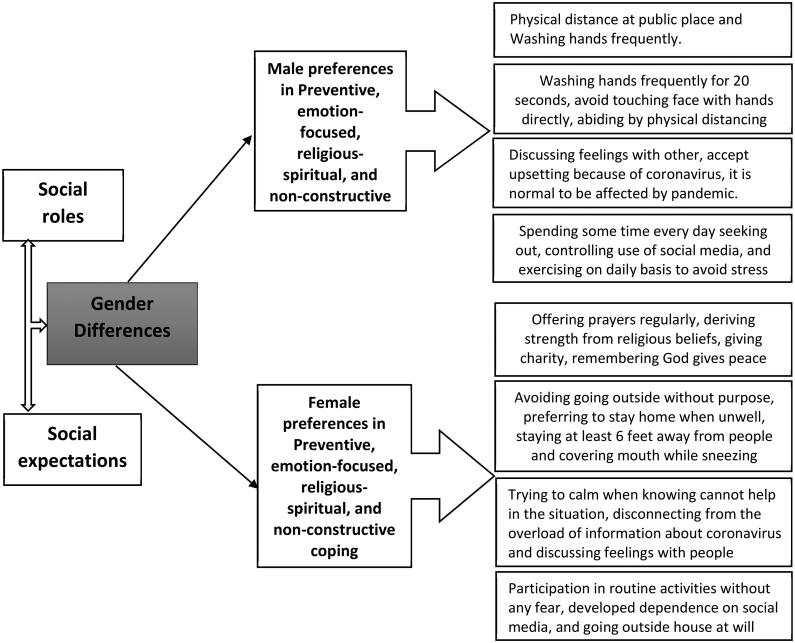
Gender differences in adoption of coping strategies.

In Pakistan, with the eruption of COVID-19, there have been higher risk perceptions among women about susceptibility and severity of the disease and the adoption of preventive coping behaviour such as social distancing and enhanced hygiene measures [23]. In addition, gender differences were notable in adopting coping strategies, with women complying with the government’s preventive instructions more than males [22]. Whereas a local study by Rana et al. [22] focused on the problem-oriented, emotion-oriented and action-oriented domains of coping, but current study will expand the realm of coping mechanisms by adding religious-spiritual and non-constructive coping. Similarly, the contextual background of Pakistan as a traditional society (with different cultural and social norms) may make it an interesting population to study. In addition, the specific gender-based socialization pattern of a traditional society can bring diversity in the adoption of coping strategies against COVID-19 (Figure 1). Existing literature highlighted that the prudence in devising coping strategies in such situations often remains highly contextual. It may be inspired by perceived levels of appropriateness and a reverence for established cultural values and social norms [24].

### Theoretical framework

Social role theory argues that the patterns in which young boys and girls are socialized into gender-based social roles impact perceptions and the construction of behaviours. Social role theory proposes that each gender employs different coping mechanisms in stressful situations [25] such as COVID-19. In this way, people internalize gender stereotypes, resulting in different coping behaviours during stressful life circumstances, such as health crises [26]. The theory also suggests that the influence of social norms generates different psychological expectations and behaviours in men and women [25]. In particular, male college students tend to show more optimism, autonomy and active behaviour in adversity (e.g. isolation, and self-quarantine during COVID-19). In contrast, women college students are more inclined toward emotion-focused coping such as being emotional, relying on others, and holding negative expectations when encountering difficulties [27,28].

Consistent with social role theory, an empirical study of gender-based differences in applying coping strategies with COVID-19 found that women tend to adopt more emotional coping (such as feeling overwhelmed, anxious, stressed, etc.) than effective coping strategies [24]. Similarly, men tend to be more effective in dealing with stress in life and perceive a lower sense of stress than women [29]. Based on social role theory and empirical evidence, this study found that gender moderated the association between the adoption of positive coping mechanisms and life-satisfaction.

### Gender and religious-spiritual coping

Religious-spiritual coping is defined as the use of religious and/or spiritual beliefs, actions, or behaviour to prevent or alleviate the unpleasant emotions elicited by stressful life circumstances [30] such as the COVID-19 pandemic. Individuals employ it for many purposes such as searching for meaning, achieving a sense of mastery and control, seeking comfort or spirituality, transformation in life, etc. [31].

Gender has been highlighted as a significant predictor of religious intensification during the COVID-19 pandemic [32]. Gender differences exist in coping mechanisms, as women have higher religious coping levels than their male counterparts during the COVID-19 pandemic [18,33]. In a country like Pakistan specifically, religion plays a vital role in people’s lives [34]. Religion and spirituality have been essential ideological tools to retain traditions and culture. For Muslim women, religious and spiritual coping has been integral in overcoming patriarchal forces and a lack of autonomy [35]. Therefore, the current study hypothesizes that women will rely more heavily on religious-spiritual coping strategies than men (H_1_).

### Gender and preventive coping

Preventive coping is like problem-focused coping, which focuses on the efforts to rectify the immediate problem [36], and it includes many actions/guidelines during the COVID-19 pandemic such as washing hands frequently, avoiding touching face/eyes/nose/mouth, avoiding going outside, wearing a mask, abiding by physical/social distancing, avoiding going to mass gatherings, etc. The basic purpose is to promote health social behaviours, and controlling the spread of the pandemic.

Past research has indicated that women exhibit better adherence to mask-wearing practices than their male counterparts [37]. During the COVID-19 pandemic, gender-predicted patterns of social distancing, avoiding mass or public gatherings, or visiting friends and relatives [17,38,39]. Similarly, a more significant proportion of women adhered to avoiding social gatherings and travel, engaging in frequent hand hygiene, and stockpiling food and medications than men [40,41]. One study the United States reported that men are more likely to perceive facemasks as infringing upon their independence while women perceive them as uncomfortable [42]. Another study the United States revealed that women perceived more threat and fear of COVID-19, which could be a reason for their comparatively better preventive practices [43]. However, one study also described how men and women are equally likely to adopt preventive coping such as wearing face masks during the COVID-19 pandemic [42]. Therefore, the current study hypothesizes that women are more likely than men to adopt preventative coping strategies (H_2_).

### Gender and emotion-focused coping

According to the theory of stress and coping, emotion-focused coping selectively focuses on positive aspects of the self and the situation (COVID-19 in the current case) by evading or recreating a stressful situation as an effort to control the emotional state related to or resulting from stress [36,44]. People use emotion-focused coping (such as wishful thinking, giving vent to their emotions, etc.) to manage or reduce their emotional stress [45]. Previous research in United Arab Emirates highlighted the inclination of females to employ a greater degree of emotion-focused coping strategies compared to men [46].

Academic scholarship reported that women demonstrated more nurturing, caregiving and sensitivity in managing pandemic stress in France [24]. Another study In the United states reported greater use of multiple emotion-focused strategies by women during the COVID-19 pandemic, including distraction, emotional and social support, and less use of humour [17]. However, a recent study in China suggests that men used more emotion-focused coping than women; Huang et al. [16] found that women were more likely to opt for problem-focused coping mechanisms (such as active coping, planning, use of instrumental support, etc.). In addition, men are more likely to opt for emotion-focused coping strategies (such as emotional support, acceptance, positive reframing, self-distraction, self-blame, denial, venting, etc.) during the COVID-19 outbreak [16]. In Turkey, in another study, emotion-focused coping was explored, but no significant association with gender was found [47]. The outlined literature highlights there is disagreement about the relationship between gender and emotion-focused coping. Given the Pakistani context, the current study expects that women are more likely than men to adopt emotion-focused coping strategies (H_3_).

### Gender and non-constructive coping

Non-constructive coping strategies entail helpless–hopeless, anxious preoccupation and fatalism about the social phenomenon [48]. In addition, it included denial, behavioural and mental disengagement, concentration on and venting of emotions, humour and substance use, etc. [49]. Academic scholarship highlighted that male participants used maladaptive coping strategies, while women used more adaptive coping [50,51].

In addition, previous literature has highlighted certain variations of non-constructive coping behaviour based on genders, such as denial and disengagement [19,42]. One study in the United States revealed the adoption of avoidance in wearing facemasks by both men and women due to independence and unease, respectively [42]. However, In United Kingdom men reported more outings during the lockdown than women [19]. Another study in China reported that both men and women had increased their use of social media since the outbreak of the COVID-19 pandemic [52]. Also, in Cyprus both men and women are committed to gaining media-based information from reliable sources [53]. Based on previous literature and the unique context of Pakistan, the current study hypothesizes that women are less likely than men to adopt non-constructive coping strategies (H_4_).

## Methods

### Ethics

Ethical approval for this study was obtained from the Ethical Review Board of the Department of Sociology, University of Gujrat, Gujrat City, Pakistan. The data collected through an online survey adopted a rigorous ethical process. The first author’s email address was provided on the informed consent page, which described the study’s objectives so respondents could contact the author if they had any feedback, or questions or needed more clarity. All the participants electronically signed informed consent.

### Sampling criterion

The target population for this study included characteristics of (i) men and women, (ii) above 18 years of age, (iii) Pakistani and (iv) users of the internet. Due to the lockdown in Pakistan, we used an online platform for data collection.

### Instrument

The variable of gender was measured using a dummy variable with women as the reference category. Out of the total 955 respondents, 52.6% were men, and 47.4% of the population were women; hence, the overall data were gender-responsive. The composite score of the variable of coping strategies was computed through values of religious-spiritual coping, preventive coping, emotion-focused coping and non-constructive coping. A five-category response Likert scale was used to measure the coping strategies such as preventive, emotion-focused, religious-spiritual and non-constructive, ranging from Never = 1, Sometimes = 2, About half of the time = 3 Most of the time = 4 and Always = 5.

#### Religious-spiritual coping

The current study modified the existing scale of religious-spiritual coping [54] according to the current needs and situation of the pandemic. As a result, the religious-spiritual coping scale has 11 items, including offering prayers regularly, praying to Allah to protect the family from the pandemic, and deriving strength from religious beliefs. In addition, believing that God will answer prayers, believing that God tests patience through such situations, seeking God’s help to recover from the pandemic, and being hopeful that God will restore the peace of the world were categories of religious-spiritual coping. Furthermore, motivating family/friends to be religious during the pandemic, giving charity to the needy, remembering God gives me peace during the pandemic, and asking for forgiveness from God were categories of religious-spiritual coping.

#### Preventive coping

The scale of preventive coping was designed following the World Health Organization guidelines related to the COVID-19 pandemic [55]. Overall, the scale of preventive coping comprised of nine items, including: washing hands frequently for at least 20 s, avoiding touching face/eyes/nose/mouth with hands, staying at least 6 ft. (2 m) away from people. In addition, covering the mouth with a tissue/inside of the elbow while sneezing or coughing, avoiding going outside without purpose, and wearing a mask while going out at work or public places were preventive coping categories. Furthermore, the categories of preventive coping were abiding by physical distancing at work or public places, preferring to stay home when not feeling well, and avoiding going to mass gatherings.

#### Emotion-focused coping

The current study modified the scale of emotion-focused coping [56] according to the current needs and situation of the pandemic. The scale of emotion-focused coping consisted of eight items, including discussing feelings with people around, acknowledging upsetting thoughts occurring because of coronavirus, reminding that scientist(s) will be able to find a vaccination for this pandemic, maintaining routine at home, understanding that it is normal to be affected emotionally by outbreaks of the pandemic and trying to calm down when knowing it cannot help in the situation, disconnecting from the overload of coronavirus information, and listening to music.

#### Non-constructive coping

The scale of non-constructive coping was constructed through literature and consisted of eight items, including participation in routine activities without any fear, not paying much attention to sources with biased coronavirus information, developing a dependence on social media to avoid the stress of coronavirus, checking the use of social media, spend some time every day seeking out information about coronavirus through reputable resources (such as news organizations, social media, etc.), and doing exercise daily to avoid the stress of this pandemic.

#### Control variables

We used control variables such as level of education, household monthly income, family structure, marital status and family size for the current study. Level of education is a categorical variable with response categories of 10 or less than 10 years (coded = 1), intermediate (coded = 2), graduation (coded = 3), Masters/BS (coded = 4), MPhil/MS (coded = 5) and Ph.D. and above (coded = 6). Similarly, household monthly income was a categorical variable with response categories of 0–30,000 PKR (coded = 1), 30,001–60,000 PKR (coded = 2), 60,001–90,000 PKR (coded = 3) and above 90,000 PKR (coded = 4). Family structure was a dichotomous variable with response categories of urban (coded = 1) and rural (coded = 2). Similarly, family structure was a dichotomous variable with response categories of joint family = 1 and nuclear family = 2. Marital status was a categorical variable with response categories of single = 1, married = 2 and divorced/separated/widowed = 3. Family size was also a categorical variable with responses of 1–4 members = 1, 5–8 members and 9 and above members = 3.

### Reliability and validity

Factor analysis and reliability analysis were performed for each of the four scales. The results for Cronbach’s alpha reliability were satisfactory for each scale: (i) religious coping scale, *α* = 0.913; (ii) preventive coping scale, *α* = 0.848 (Supplementary Table 8); (iii) emotion-focused coping scale, *α* = 0.731; and (iv) non-constructive coping scale, *α* = 0.631.

### Data collection

The questionnaire was constructed through the Qualtrics Survey Design website in both English and Urdu language for the convenience of the respondents. Pretesting was done with 30 respondents not included in the final analysis. The primary purpose of the pre-testing was to identify problems with the data collection tool. Based on feedback from pilot-test respondents, the survey improved for wording, readability and sentence structuring. The final questionnaire for the current study was shared on different social media groups, such as Twitter, Facebook and WhatsApp. The data were collected during the first wave of the COVID-19 pandemic between 17 May 2020 and 25 October 2020. The survey reached 1035 individuals, and 955 participated and completed the survey. The 80 people (7.7%) who did not participate in the study either: (i) did not fill the inclusion criterion or (ii) were not willing to participate after reading the study objectives.

### Data analysis

We used Stata/SE 17.0 (StataCorp, College Station, TX) and Statistical Package for Social Sciences (SPSS) version 26 (SPSS Inc., Chicago, IL) for data analysis. Factor analysis was performed to find out the validity of the categories of the scales, along with reliability analysis to verify the internal consistency of the scales. Finally, regression was calculated to identify the association between gender and coping strategies. We used two models; model 1 was based on the association between dependent and independent variables, while model 2 explored association in the presence of control variables. To test the overall model fit, binary logistic regression analysis was executed. Three different levels of significance were assigned at *p* < .05 (weak evidence), *p* < .01 (moderate evidence) and *p* < .001 (good evidence).

## Results

### Socio-demographic results

Table 1 represents the descriptive statistics of socio-demographic variables. In gender, 52.6% were males while 47.4 were females. In age, 80.6% were in the age bracket of 18–27 years, 15.3% were in the age group of 28–37 years and only 4.0% were 38 years and above. In education, 0.9% had 10 or fewer years of education, 10.8% had an intermediate level of education, 27.9% had a graduation level of education, 39.5% had master’s or BS honors, 18.0% had Master of Philosophy and 2.9% had Ph.D. In monthly household income, 34.3% have 1–30,000 Pakistani Rupees (PKR), 27.2% have 30,001–60,000 PKR monthly household income, 15.5% had 60,001–90,000 PKR monthly household income, while 23.0 had above 90,000 PKR as monthly household income. In the residential background, 53.7% were living in urban areas, while 46.3 were living in rural areas. 53.2% lived in a joint family system in the family structure, while 46.8% lived in nuclear families. In marital status, 80.1% were single, 18.6 were married and 1.2% were divorced, separated or widowed. In family size, 21.9% had 1–4 family members, 54.5% had 5–8 family members and 23.6% had nine and more family members.

**Table 1.** Descriptive statistics of demographics of the respondents (*N* = 955).

Categories	Frequency	Percentage
Gender		
Male	502	52.6%
Female	453	47.4%
Age		
18–27 years	770	80.6%
28–37 years	146	15.3%
38 years and above	39	4.0%
Education		
10 or less than 10 years	09	00.9%
Intermediate	103	10.8%
Graduation	266	27.9%
Masters/BS	377	39.5%
MPhil/MS	172	18.0%
Ph.D. and above	28	02.9%
Monthly household income		
30,000–60,000 PKR	260	61.5%
60,001–90,000 PKR	148	15.5%
Above 90,000 PKR	219	23.0%
Residential background		
Urban	513	53.7%
Rural	442	46.3%
Family structure		
Joint family	508	53.2%
Nuclear family	447	46.8%
Marital status		
Single	765	80.1%
Married	178	18.6%
Divorced/separated/widowed	12	01.2%
Family size		
1–4 members	209	21.9%
5–8 members	520	54.5%
9 and above	226	23.6%

### Descriptive results

Table 2 reports descriptive statistics for gender and religious-spiritual coping strategies. We found overall that women reported higher religious-spiritual coping strategies such as offering prayers regularly (61.4% vs. 50.4%), deriving strength from religious beliefs (83.4% vs. 69.1%), giving charity to the needy during this pandemic (72% vs. 63%) and remembering God gives peace during pandemic (77% to 71.1%) as compared to men. While the gender differences were not higher in being hopeful God will restore the peace of the world (86.1% vs. 83.1%), praying to Allah to protect the family from this COVID-19 pandemic (83.4% vs. 80.1%), seeking God’s help to recover from the coronavirus pandemic (86.1% vs. 83.1%)), motivating family/friends to be religious during the COVID-19 pandemic (73.5% vs. 71.8%) and asking for forgiveness to God (87.7% vs. 86.7%) between males and females.

**Table 2.** Descriptive statistics showing religious-spiritual coping by gender.

Categories of religious-spiritual coping	Females (453)		Males (502)	
	Never/sometimes/about half of times	Most of the time/always	Never/sometimes/about half of the times	Most of the time/always
I offer prayers regularly	175 (38.6%)	278 (61.4%)	249 (49.6%)	253 (50.4%)
I pray to Allah to protect me/my family from this pandemic	75 (16.6%)	378 (83.4%)	100 (19.9%)	402 (80.1%)
I derive strength from my religious beliefs	101 (22.3%)	352 (77.7%)	155 (30.9%)	347 (69.1%)
I believe that God will answer my prayers	67 (14.8%)	386 (85.2%)	103 (20.5%)	398 (79.5%)
I believe, God tests patience through a such pandemic situation	74 (16.3%)	379 (83.7%)	110 (21.9%)	392 (78.1%)
I seek God’s help to recover from the coronavirus pandemic	63 (13.9%)	390 (86.1%)	85 (16.9%)	417 (83.1%)
I am hopeful God will restore the peace of the world	34 (7.5%)	419 (92.5%)	56 (11.1%)	446 (88.9%)
I motivate my family/friends to be religious during the pandemic	120 (26.5%)	333 (73.5%)	142 (28.2%)	360 (71.8%)
I give charity to the needy during this pandemic	172 (38.0%)	281 (72.0%)	186 (37.0%)	316 (63.0%)
Remembering God gives me peace during the pandemic	104 (23%)	349 (77%)	145 (28.9%)	357 (71.1%)
I ask for forgiveness to God	56 (12.3%)	397 (87.7%)	67 (13.3%)	435 (86.7%)

Table 3 reports the descriptive statistics for gender and preventive coping. We found overall that women reported higher preventive coping as compared to men in the following areas: (i) voiding going outside without purpose (81.2% vs. 69.1%); (ii) preferring to stay home when not well (78.8% vs. 69.1%); (iii) staying at least 6 ft. away from people (42.4% vs. 35.1%); and (iv) covering mouth with a tissue/inside of elbow while sneezing or coughing (78.1% vs. 69.1%). In contrast, men reported slightly higher preventive coping strategies, such as washing hands frequently for at least 20 s (39.1% vs. 69.5%), avoiding touching face/eyes/nose/mouth with hands directly (54.5% vs. 58.4%) and abiding by physical distancing at work or public places women (67.6% vs. 72.2%).

**Table 3.** Descriptive statistics of cross-tabulation of gender and preventive coping.

Categories of preventive coping	Females (453)		Males (502)	
	Never/sometimes/about half of times	Most of the time/always	Never/sometimes/about half of the times	Most of the time/always
I wash my hands frequently for at least 20 s	139 (30.7%)	314 (39.1%)	153 (30.5%)	349 (69.5%)
I avoid touching my face/eyes/nose/mouth with my hands directly	209 (45.5%)	244 (54.5%)	209 (41.6%)	293 (58.4%)
I stay at least 6 feet (2 meters) away from people.	261 (57.6%)	192 (42.4%)	326 (64.9%)	175 (35.1%)
I cover my mouth with a tissue/inside of the elbow while sneezing or coughing.	99 (21.9%)	354 (78.1%)	155 (30.9%)	347 (69.1%)
I avoid going outside without purpose.	85 (18.8%)	368 (81.2%)	150 (29.9%)	352 (69.1%)
I wear a mask while going out at work or in public places.	54 (11.9%)	399 (88.1%)	104 (20.7%)	398 (79.3%)
I abide by physical distancing at work or in public places.	147 (32.4%)	306 (67.6%)	190 (37.8%)	312 (72.2%)
When I am not feeling well, I will prefer to stay home	96 (21.2%)	357 (78.8%)	155 (30.9%)	347 (69.1%)
I avoid going to mass gatherings	95 (21.0%)	358 (79.0%)	143 (28.5%)	359 (71.5%)

Table 4 reports descriptive statistics for gender and emotion-focused coping strategies. We found overall that women reported higher emotion-focused coping, such as trying to calm down when knowing cannot help in the situation (67.7% vs. 57.8%), disconnecting from the overload of information about coronavirus (59.2% vs. 53.6%) and discussing feelings with people around (30.7% vs. 24.9%) as compare to men. Men scored slightly higher on following (i) discussing ­feelings with people around (50.3% vs. 51.2%), acknowledging that upsetting thoughts occurring because of the coronavirus (42.8% vs. 44.2%) although the differences were minute. Similarly in many other emotion-focus coping strategies such as reminding myself that scientist(s) will be able to find a vaccination for this pandemic (56.1% vs. 55.4%) and understanding that it is normal to be affected emotionally by pandemic outbreaks (65.4% vs. 64.3%), the differences were minor among men and women.

**Table 4.** Descriptive statistics of cross-tabulation of gender and emotion-focused coping.

Categories of emotion-focused coping	Females (453)		Males (502)	
	Never/sometimes/about half of times	Most of the time/always	Never/sometimes/about half of the times	Most of the time/always
I discuss my feelings with people around me	225 (49.7%)	228 (50.3%)	245 (48.8%)	257 (51.2%)
I acknowledge my upsetting thoughts occurring because of the coronavirus	259 (57.2%)	194 (42.8%)	280 (55.8%)	222 (44.2%)
I remind myself that scientist(s) will be able to find a vaccination for this pandemic	199 (43.9%)	254 (56.1%)	224 (44.6%)	278 (55.4%)
I maintain my routine at home as much as I can.	128 (28.3%)	325 (71.7%)	159 (31.7%)	343 (68.3%)
I understand that it is normal to be affected emotionally by pandemic outbreaks.	157 (34.6%)	296 (65.4%)	179 (35.7%)	323 (64.3%)
I try to calm myself when I know I cannot help in the situation.	151 (33.3%)	302 (67.7%)	212 (42.2%)	290 (57.8%)
I disconnect myself from the overload of coronavirus information.	185 (40.8%)	268 (59.2%)	233 (46.4%)	269 (53.6%)
I listen to music to avoid my attention	244 (53.9%)	209 (46.1%)	288 (57.4%)	214 (42.6%)
I discuss my feelings with people around me	314 (69.3%)	139 (30.7%)	377 (75.1%)	125 (24.9%)

Table 5 reports the descriptive statistics for non-constructive coping. Overall, men reported higher on non-constructive coping strategies, such as spending some time every day seeking out (31.3% vs. 37.1%) information about coronavirus through reputable resources such as news organizations, social media, etc. (37.5% vs. 39.7%), controlling the use of social media, paying much attention to sources with biased information on coronavirus (48.8% vs. 62.0%), and exercising daily to avoid the stress of pandemic (30.5% vs. 37.9%) as compared to women. while in many other non-coping strategies such as participating in routine activities without any fear (62.7% vs. 61.2%), developing a dependence on social media to avoid the stress of the coronavirus (44.8% vs. 43.4%) and going outside the house at will (39.1% vs. 35.7%), women reported higher but the differences were very minor.

**Table 5.** Descriptive statistics showing non-constructive coping by gender.

Categories of non-constructive coping	Females (453)		Males (502)	
	Never/sometimes/about half of times	Most of the time/always	Never/sometimes/about half of the times	Most of the time/always
I participate in routine activities without any fear	169 (37.3%)	284 (62.7%)	195 (38.8%)	307 (61.2%)
I have developed a dependence on social media to avoid the stress of the coronavirus	250 (55.2%)	203 (44.8%)	285 (56.7%)	217 (43.4%)
I do not wear a face mask	379 (75.5%)	74 (24.5%)	421 (83.9%)	81 (16.1%)
I go outside my house at my will	276 (60.9%)	177 (39.1%)	323 (64.3%)	179 (35.7%)
I spend some time every day seeking out information about coronavirus through reputable resources (such as news organizations, social media, etc.)	311 (68.7%)	142 (31.3%)	316 (62.9%)	186 (37.1%)
I have controlled my use of social media.	283 (62.5%)	170 (37.5%)	303 (60.3%)	199 (39.7%)
I am not paying much attention to sources with biased coronavirus information.	232 (51.2%)	221 (48.8%)	291 (58.0%)	211 (62.0%)
I exercise on daily basis to avoid the stress of this pandemic	315 (69.5%)	138 (30.5%)	313 (62.1%)	189 (37.9%)

### Regression analysis

Table 6 reports the regression analysis between gender and the four coping strategies measured in this study. Model 1 highlighted the association between gender and different coping strategies (such as religious-spiritual coping, preventive coping, emotion-focused coping and non-constructive coping). Model 2 highlighted the association between gender and different coping strategies in the presence of some control variables such as level of education, household monthly income, family structure, marital status and family size. Results revealed that there was a positive significant association between gender and religious-spiritual coping, which means religious-spiritual coping was higher among females as compared to males. The value of the odd ratio suggested that the adoption of religious-spiritual coping was 84% higher among females as compared to males. In model 2, still there was significance between gender and religious-spiritual coping but the association became weak in the presence of control variables. In the presence of control variables, the adoption of religious-spiritual coping was 41% higher among females as compared to males. Similarly, there was a positive significant association between gender and preventive coping, which means preventive coping was higher among females as compared to males. The value of the odd ratio suggested that the adoption of preventive coping was 98% higher among females as compared to males. In the model, still there was an association between gender and preventive coping, which was 62% higher among females as compared to males in the presence of control variables.

**Table 6.** Regression analysis between gender identity and adoption of coping strategies against COVID-19.

Types of coping strategies	Model 1	Model 2
Religious-spiritual coping	1.841**	1.41*
Preventive coping	1.978***	1.62**
Emotion-focused coping	.948*	.75
Non-constructive coping	−0.283	−0.49
Coping strategies	4.535**	3.36*

*= *p* <.05; **= *p* <.01; ***= *p* <.001.

On the contrary, there was an association between gender and emotion-focused coping in model 1, which vanished with the introduction of control variables in model 2. There was no association between gender and non-constructive coping with and without the presence of the control variables. Finally, there was a positive association between gender and coping strategies, which stated that coping strategies were 4.5 times higher among females as compared to males. With the introduction of control variables, the association became weak but still, the coping was 3.4 times higher among females as compared to males.

### Binary logistic regression analysis

Table 7 highlights the descriptive statistics of binary logistic regression analysis between gender identity and various coping strategies such as religious-spiritual, preventive, emotion-focused, non-constructive, etc. The value of the *p < .001* suggested an overall model fit. In the overall model, religious-spiritual coping (*odd ration = 1.02, p < .05*), and preventive coping (*standardized beta = 1.03, p < .01*), were influenced by gender identity. Women experienced a 2% increase with one unit increase in religious-spiritual coping strategies and experienced a 3.3% increase with one unit increase in preventive coping as compared to men.

**Table 7.** Binary logistic regression analysis of gender identity with various coping strategies against COVID-19.

Gender	Odd ratio	Std. err.	*z*	*p* > *z*	95% CI
Religious-spiritual coping	1.015861	.0074791	2.14	.033	1.001307–1.030626
Preventive coping	1.033474	.0118828	2.86	.004	1.010445–1.057029
Emotion-focused coping	.9992659	.0126589	−0.06	.954	.9747604–1.024387
Non-constructive coping	.9823682	.0120106	−1.46	.146	.9591077–1.006193
Constant	.2124207	.0960613	−3.43	.001	.0875517–.5153816

LR chi2(4) = 22.94, Prob > chi^2^ = 0.0001, Pseudo *R*^2^ = 0.0174.

The positive values of the standardized beta suggested that women were more likely than men to adopt religious-spiritual and preventive coping strategies during the pandemic. Contrarily, emotion-focused and non-constructive coping strategies had no association with gender identity in an overall model (see Table 7).

## Discussion

Findings highlighted that women reported higher religious-spiritual coping such as offering prayers regularly, deriving strength from religious beliefs, giving charity to the needy during this pandemic, and remembering God gives peace during the pandemic. Similarly, a recent study in Morocco highlighted the use of religiosity, and charity during COVID-19 [57]. In addition, based on findings from the Netherlands, a study stated that stimulating existing religious and spiritual resources may enable persons to function better in case of pandemics and other collective stressors [58]. The current study reported higher preventive coping such as avoiding going outside without purpose, preferring to stay home when not well, staying at least 6 ft. away from people, and covering mouth with tissue/inside of elbow while sneezing or sneezing coughing. Furthermore, they reported higher emotion-focused coping, such as trying to calm down when knowing cannot help in the situation, disconnecting from the overload of coronavirus information, and discussing feelings with people around. On the contrary, men reported higher on washing hands frequently for at least 20 seconds, avoiding touching their face/eyes/nose/mouth with their hands directly, abiding by physical distancing at work or public places, spending some time every day seeking out information about coronavirus through reputable resources such as news organizations, social media, etc., controlling the use of social media, paying much attention to sources with biased coronavirus information, and exercising daily to avoid the stress of this pandemic.

Findings reveal that Pakistani women have higher adoption of coping strategies than their male counterparts during the COVID-19 pandemic for religious-spiritual, preventive and emotion-focused coping. Similar differences based on sex in the adoption of religious coping were highlighted by the research conducted in Jordan during the COVID-19 pandemic [59]. A study from Turkey highlighted the difference among adolescents as females tend to use positive religious coping while males use negative religious coping and the differences might be because of God as controlling is higher among men [60]. Study findings contradict with the previous research in India and Nigeria that identified no significant difference in adoption of religious-spiritual coping based on gender [61]. However, the results in this study for higher religious-spiritual coping among women, compared to men, corroborated findings from United States and Poland [62,63]. Similarly, women reported higher use of religious coping as compared to men in Saudi Arabia [64]. Furthermore, the results imply that women from Pakistan may be influenced during times of crises and hardships due to their higher commitment to participation in religious events and activities and higher patterns of religiosity [65–67].

Women compared to men reveal higher scores for preventive coping, specifically for mask-wearing, isolation when not well, and avoidance of mass gatherings. The findings corroborate with recent studies, confirming that women adopt more preventive strategies than men during the coronavirus pandemic [43]. It may be because men are more likely to perform risky health behaviours, like avoiding facemasks and attending public gatherings. After all, they have been socialized to exhibit independence and maintain networking in public gatherings for work or family traditions [42,68]. Previous research has also reinforced the association between masculinity and risky health behaviours [69,70]. Previous studies in Australia [71], the United States [72] and Belgium [73] have also highlighted that higher adoption of preventing coping among women might have to do with higher rates of distress and fear of the COVID-19 pandemic. More research is needed to confirm that women may be adopting higher coping strategies due to more significant stress and fear. If this is true, a critical policy would need to be developed to support women for psychological support.

The research also found a higher association between women and emotion-focused coping strategies. Females scored higher on emotion-focused coping, which highlighted gender differences in a study conducted in Croatia [74]. Conversely, the findings contradict a previous study in China, which reports that women are less likely to choose emotion-focused coping strategies during the COVID-19 pandemic [16]. Instead, our findings support recent research in France and Pakistan, highlighting that women demonstrate more significant nurture, care and sensitivity, in the face of the anxiety caused by the pandemic [22,24]. Our findings confirmed the findings of a previous study in Israel, which stated that women used more coping tactics, both emotion-focused and problem-focused [75]. Women in traditional societies may have been socialized from the early years to use more emotions and care in times of crises and illness [29,36,76]. Women may also be more aware of their emotions and are more willing to manifest their concerns and care instincts than men [17,24,77]. Increased emotion-focused coping among females can also be a sign of controlling the distress induced by the COVID-19 pandemic [46], where they might be using emotion-focussed coping as a protective factor against the public health emergency. Similarly, in a patriarchal society like Pakistan, the accepted cultural expectation that female gender identity is connected to intuitive and emotional style coping mechanisms and cumulative burden for everyday stressors can be another rationale for these differences in coping mechanisms [78]. Ultimately, we must also consider that women who use more emotion-focused coping may also require support due to increased emotional burdens during and after the pandemic. This is also corroborated by literature, which stated that females reported significantly elevated levels of stress and psychological problems, such as depression and anxiety during the current COVID-19 pandemic [4,79], and emotion-focused coping reduces negative emotions associated with the COVID-19 pandemic as the stressor [74]. Men use more problem-focused coping while women use more emotion-focused coping and social support seeking [80]. Our finding corroborated with findings related to emotion-focused coping while contradicting problem-focused coping as females reported higher emotion-focused and preventive coping as well.

Overall, the adoption of coping strategies was four times higher among females as compared to males, which highlighted obvious gender differences in the adoption of coping strategies during COVID-19. From the psychological symptomology point of view, these gender differences indicate that psychological reactions may be due to the heavier toll taken by the pandemic and lockdown on the lives of women rather than to gender differences in coping tactics or resilience [75]. In addition, there may be many justifications for these gender-based differences. For example, literature highlighted that gender differences may be because of differences in perception of the COVID-19 pandemic, which ultimately can influence individuals of different genders to respond differently [81].

### Study limitations, implications and future research

Like other studies, the present study also has some limitations. First, the findings cannot be generalized to the Pakistani public because the target population has relatively higher education. All the respondents were social media users, so maybe they had been relatively more exposed and had higher knowledge of the COVID-19 pandemic than others because of the context of a social-media environment that rapidly connects the population to ongoing messages about the spread and ramifications of the virus. Furthermore, as data were collected during the lockdown in the country, the government institutions’ restrictions and guidelines might have influenced the overall score of preventive coping. Finally, the data were collected during a COVID-19 pandemic, so its findings of gender differences in adopting coping strategies cannot be generalized to non-pandemic situations.

The empirical evidence suggested that females might be at an increased risk of stress due to the burden of unbalanced household-based social norms and care responsibilities. The current research also expanded the base of coping to religious-spiritual coping, emotion-focused coping and non-constructive coping. Although the World Health Organization announced the end of the COVID-19 pandemic Public Health Emergency, still COVID-19 pandemic raises its head in different parts of the world and some places also impose lockdowns. Having said that, the empirical evidence of the current study can be relevant to its coping. In addition, these findings can also serve as guidelines for the policymakers for future pandemics.

Future research can analyse whether marital or parental status affects coping behaviours for men and women. If this point is valid, it would be expected that married women and women with children would adopt more preventative strategies than single or childless women. More research in this area is needed to develop and support women’s active and productive role within the household to promote public health and positive coping strategies for families during times of crisis. In addition, future research can enhance the base of the individual and personality traits (such as age, level of education, income, etc.) in the adoption of different coping strategies (such as religious-spiritual, preventive or problem-solving, emotion-focussed, and non-constructive coping, etc.) during the COVID-19 pandemic so that it becomes easy for policymakers to design policies that reach out to the vulnerable population needing emergency socio-medico-psychological support and other resources. Finally, it will be interesting to know how cultural implications (such as social pressure, labelling, etc.) and the occurrence of the perceived stigma of COVID-19 can influence the adoption of different coping mechanisms among the public while mediating the role of exposure and knowledge of the COVID-19 pandemic. Future research can also find how the adoption of coping mechanisms can interact with mental health-related issues (such as depression, anxiety, loneliness, sleeplessness, etc.). Future research can also come up with a comparative analysis (whether cross-sectional or longitudinal, etc.) of the effectiveness of different coping strategies during pandemics.

## Conclusions

Findings highlighted that women reported higher religious-spiritual coping (such as offering prayers regularly, deriving strength from religious beliefs, giving charity and remembering God give peace during the COVID-19 pandemic), preventive coping strategies (such as avoiding going outside without purpose, preferring to stay home when not well, staying at least 6 ft. away from people, etc.) and emotion-focused coping strategies (such as trying to calm down when knowing cannot help in the situation, disconnecting from the overload of coronavirus information, and discussing feelings with people around, etc.). On the contrary, men reported higher non-constructive coping (such as spending some time every day seeking out information about coronavirus through reputable resources such as news organizations, social media, etc., controlling the use of social media, paying much attention to sources with biased coronavirus information, and exercising daily to avoid the stress of this pandemic, etc.).

The current study revealed that women were more likely to adopt most coping strategies, with the most significant difference in preventative coping strategies. There was no gender difference in adopting non-constructive strategies. In times of major upheavals and unforeseen catastrophes such as the COVID-19 pandemic, gender-based social roles and responses can change meaning and utility. The COVID-19 pandemic has augmented the adoption of coping strategies in Pakistani women, compared to men, concerning religious-spiritual coping, preventative coping and emotion-focused coping. As a result, there is concern that females in the country might be at increased risk of stress and emotional fatigue due to their coping behaviours. However, findings also imply that women can play a crucial role in promoting preventive behaviour within the family unit while adopting and even advocating positive spirituality and emotional support practices.

## Supplementary Material

Supplemental Material

## Data Availability

Data will be available from the corresponding author upon a reasonable request.
