# Supplementary material for: Influence of gender identity on the adoption of religious-spiritual, preventive and emotion-focused coping strategies during the COVID-19 pandemic in Pakistan
Source: Ann Med. 2023 Dec 17;55(2):2291464. doi: 10.1080/07853890.2023.2291464 (PMC10732200; doi:10.1080/07853890.2023.2291464)
Supplement: Supplemental Material [file IANN_A_2291464_SM0042.docx]

# Supplementary File

**Table 8: Factors analysis of religious-spiritual coping**

| Categories of religious-spiritual coping (value of Cronbach alpha=0.913) | Component |
| --- | --- |
|  | 1 |
| I offer prayers regularly | .604 |
| I pray to Allah to protect me/my family from this pandemic | .675 |
| I derive strength from my religious beliefs | .749 |
| I believe that God will answer my prayers | .826 |
| I believe God tests patience through such a pandemic situation | .828 |
| I seek God's help to recover from the coronavirus pandemic | .866 |
| I am hopeful God will restore the peace of the world | .836 |
| I motivate my family/friends to be religious during the pandemic | .722 |
| I give charity to the needy during this pandemic | .498 |
| Remembering God gives me peace during the pandemic | .762 |
| I ask for forgiveness from God | .798 |

**Table 9: Factor analysis of preventive coping**

| Categories of preventive coping (value of Cronbach alpha=0.848) | Component | |
| --- | --- | --- |
|  | 1 | 2 |
| I wash my hands frequently for at least 20 seconds | .615 | .497 |
| I avoid touching my face/eyes/nose/mouth with my hands directly | .474 | .713 |
| I stay at least 6 feet (2 meters) away from people. | .583 | .117 |
| I cover my mouth with a tissue/inside of the elbow while sneezing or coughing. | .667 | .214 |
| I avoid going outside without purpose. | .743 | -.201 |
| I wear a mask while going out at work or in public places. | .744 | -.148 |
| I abide by physical distancing at work or in public places. | .787 | -.208 |
| When I am not feeling well, I will prefer to stay home | .663 | -.384 |
| I avoid going to mass gatherings | .763 | -.232 |

**Table 10: Factors analysis of emotion-focused coping**

| Categories of emotion-focused coping (value of Cronbach alpha=0.731) | Component | |
| --- | --- | --- |
|  | 1 | 2 |
| I discuss my feelings with people around me | .609 | -.054 |
| I acknowledge my upsetting thoughts occurring because of coronavirus | .505 | -.433 |
| I remind myself that scientist(s) will be able to find a vaccination for this pandemic | .516 | -.037 |
| I maintain my routine at home as much I can. | .642 | -.146 |
| I understand that it is normal to be affected emotionally by pandemic outbreaks. | .761 | -.068 |
| I try to calm myself when I know I cannot help in the situation, | .791 | -.019 |
| I disconnect myself from the overload of coronavirus information. | .601 | .248 |
| I listen to music to avoid my attention | .332 | .854 |

**Table 11: Factors analysis of non-constructive coping**

| Categories of non-constructive coping (value of Cronbach alpha=0.631) | Component | |
| --- | --- | --- |
|  | 1 | 2 |
| I participate in routine activities without any fear | .463 | -.342 |
| I have developed a dependence on social media to avoid the stress of coronavirus | .450 | -.162 |
| I do not wear a face mask | .565 | -.469 |
| I go outside my house at my will | .629 | -.437 |
| I spend some time every day seeking out information about coronavirus through reputable resources (such as news organizations, social media, etc.) | .516 | .489 |
| I have controlled my use of social media. | .517 | .563 |
| I am not paying much attention to sources with biased coronavirus information. | .529 | .004 |
| I do exercise daily to avoid the stress of this pandemic | .552 | .409 |
